# Identification of beneficial Lebanese *Trichoderma* spp. wheat endophytes

**DOI:** 10.3389/fpls.2022.1017890

**Published:** 2022-12-02

**Authors:** Naeif Matar, Catherine Macadré, Gamal A. G. Ammar, Alexis Peres, Boris Collet, Naim El Boustany, Loïc Rajjou, Falah As-Sadi, Marie Dufresne, Pascal Ratet

**Affiliations:** ^1^ Université Paris-Saclay, CNRS, INRAE, Univ Evry, Institute of Plant Sciences Paris-Saclay (IPS2), Orsay, France; ^2^ Université de Paris, Institute of Plant Sciences Paris-Saclay (IPS2), Orsay, France; ^3^ Department of Life & Earth Sciences, Faculty of Sciences I, Laboratory of Microbiology, Lebanese University, Beirut, Lebanon; ^4^ Biotechnology Unit, Plant Production Department, Arid Lands Cultivation Research Institute, City of Scientific Research and Technological Applications, Alexandria, Egypt; ^5^ Université Paris-Saclay, INRAE, AgroParisTech, Institut Jean-Pierre Bourgin (IJPB), Versailles, France; ^6^ Department of Biology, Faculty of Sciences I, Lebanese University, Beirut, Lebanon; ^7^ The Lebanese University, Faculty of Agronomy, Beirut, Lebanon

**Keywords:** auxins, 3-indole-acetic acid, plant root growth promotion, wheat yield, antifungal activity

## Abstract

Wheat is one of the most important crops in the world. Its production can be influenced by a diversity of beneficial and pathogenic rhizospheric microbes, including fungi. Amongst them, beneficial *Trichoderma* spp. can be used as alternatives to chemical fertilizers, as they are cheap and harmless to the environment. Our study aimed to isolate, identify, and characterize *Trichoderma* spp. from Lebanon associated with wheat. Two *Trichoderma* strains belonging to *T. afroharzianum*, and *T. guizhouense* species, were isolated and found to be endophytes, enhancing root growth and producing Indole-3-acetic acid. Inoculation also improved seedling development, and increased plant growth and yield. Furthermore, the two strains inhibit Fusarium growth *in vitro*. These *Trichoderma* spp. have thus the capacity to be used as organic fertilizers for wheat.

## Introduction

Wheat (*Triticum* sp. L.) is one of the most important crops in the world, ranking third behind corn and rice, while providing the basic nutrients (carbohydrates, proteins, vitamins, minerals, and fibers) to humans (Asseng et al., 2011). The world population is projected to exceed 8 billion by the end of 2022, and the demand for wheat is expected to exceed 880 million metric tons by 2050 (https://www.fao.org/worldfoodsituation/csdb/en/). Wheat culture, therefore, needs to be widened to overcome the world’s increasing demand. In addition, recent challenging crises, like the Covid-19 (coronavirus disease 2019) pandemic, and world conflicts negatively impact wheat production and will probably threaten wheat markets in the near future (Glauben et al., 2022; ASCAME, 2022). On the other hand, communities are requesting sustainable, cleaner, and harmless production approaches to avoid more climate change development. In this context, alternative strategies have to be followed, and finding new safer solutions is necessary in order, for example, to reduce the widespread use of chemical fertilizers, which negatively affect earth’s natural resources (Kannojia et al., 2017). As a result, recent studies have aimed to improve biofertilizers and plant bio stimulants using beneficial bacteria, yeasts, or fungi (Berruti et al., 2016; Kashyap et al., 2021). Among fungi, *Trichoderma* spp. are ubiquitous free-living fungi (Esposito and da Silva, 1998; Almi and Dehimat, 2016) of the *Hypocreaceae* family, occurring widely in all soils (Ghazanfar et al., 2018) and representing major components of the soil mycoflora (Widden and Abitbol, 1980; Kubicek et al., 2003). These ascomycete fungi, which inhabit root ecosystems, can grow and proliferate using carbohydrates secreted by plant roots. They can also behave as endophytes, penetrating the epidermis of the root tissue (rhizodermis), and few cell layers below (Topolovec-Pintarić, 2019). They proliferate better at mesophilic temperatures (15–35° C) (Carro-Huerga et al., 2021) and tolerate a wide pH range (Topolovec-Pintarić, 2019). These fungi improve plant photosynthetic capacity, and induce defenses (Vargas et al., 2011; Manzar et al., 2021) and many stress-sensitive genes (Brotman et al., 2012; Kashyap et al., 2022). *Trichoderma* are among the best studied biological control microbes, and are marketed as active ingredients in biofertilizers (plant growth promoters), biopesticides, bioremediates, and natural resistance stimulants. Consequently, their application reduces production costs, and negative environmental impacts (Dennis and Webster, 1971a; Dennis and Webster, 1971b; Harman and Kubicek, 2002; Ghazanfar et al., 2018). *Trichoderma* fungi improve plant growth through the solubilization of nutrients, and therefore have better effects under nutritional and abiotic stress conditions (Bononi et al., 2020; Chen et al., 2021). The use of *Trichoderma* in seed coating also improves physiological stresses such as aging, and seed dormancy (Delgado-Sánchez et al., 2010; Mastouri et al., 2010; Delgado-Sánchez et al., 2011). *Trichoderma* is also able to produce auxin, a plant hormone that controls many aspects of development including plant tissue growth, cell division, cell differentiation, and protein synthesis.

In this study we identified and characterized two *Trichoderma* strains from wheat plants and rhizosphere sampled in Lebanon. We then tested their capacity to behave as endophyte as well as their ability to promote wheat growth and yield.

## Materials and methods

### Plant material

The wheat cultivar ‘Apogee’ was used for fungal characterization (Li et al., 2017; Gatti et al., 2018). Apogee has a relatively brief life cycle under long days, and without vernalization, it flowers 25 days after planting.

### Soil and plant sampling

Samples in this study were collected on May 2020 (late spring and early summer) from three different sites in Lebanon, namely: 1) The international center for agricultural research in the dry areas (ICARDA) -Terbol; 33.81263953421374, 35.99166470541601, 2) Lebanese agricultural research institute (LARI) – Tal Amara; 33.86500666714277, 35.984715713142634, and 3) Doueir; 33.39043029381532, 35.417987723093724. For every location, five samples from soil, together with five plant samples were screened for *Trichoderma* spp.

Soil samples were taken from the soil section called “horizon”, using a 0 to 15 cm top layer shovel, representing the surface area of the profile, where nutrients and most of the roots are present, and where nutrient absorption takes place. This also corresponds to the area where *Trichoderma* spp. are generally found. Sampling was carried out within a radius of 10 to 30 cm from the base of the plant depending on the case. Each sample was transferred in an appropriate plastic bag with its reference (place, date, sample number, etc.). Five samples were collected, and homogenized before the final packaging of approximately 1 kg of the sample, placed in plastic bags (to avoid contamination) was kept frozen (-20°C).

For plant sampling, 5-6 independent plant samples (root and stem) spaced at least 10 m apart were placed in plastic bags and kept frozen (-20°C).

### Fungus isolation from soil

1g of soil was homogenized in 10 ml sterile distilled water by thorough vortexing, then filtrated onto sterile Miracloth^®^. The filtrate was then plated onto 9 cm diameter petri dishes (100 ul filtrate plated per dish), containing potato dextrose agar (PDA) amended with rose Bengal (0.15 g/L), pentachloronitro-benzene (0.15 g/L), chloramphenicol (34 µg/ml), and ampicillin (100 µg/ml). One petri dish was plated per soil mixture. Plates were incubated at 26°c in the dark for 4 days

### Fungus isolation from plants

The internal colonization of roots or stems by potential *Trichoderma* spp. was detected following surface sterilization of approximately 2 cm root fragments for 10 sec in 95% ethanol, rinsed in sterile water for 10 sec, submerged for 20 sec in 2.5% sodium hypochlorite, then washed three times (2 times for 20 sec, and a third time for 60 sec) in sterile water. Fragments were then left to dry under laminar flow on sterile filter. 0.5 cm fragments were placed on PDA amended with rose Bengal, pentachloronitro- Benzene (0.15 g/L), chloramphenicol (34 µg/ml) and ampicillin (100 µg/ml). Plates were incubated at 24°C in the dark for 4-6 days, until fungal development. After 4 days, a large number of fungal strains appeared and were transferred (incubated at 26°C). Later, a 10-day-old, pre-incubated Petri dish (26°C) was selected, in order to make the 2nd transfer on PDA complemented with Rose Bengal (for Soil and Plant samples). A week after, purification was done by scraping half a colony, depending on the growth rate. Purification was done by plating 1/10, 1/100 or 1/1000 dilutions on PDA complemented with Rose Bengal. After 7 days of fungal development at 26°C, pieces of the grown fungus were transferred each into new Petri dish containing PDA media and again incubated at 26°C for further development.

### DNA isolation, sequencing and phylogenetic analyses

*DNA isolation*: Liquid culture were done in potato dextrose broth (PDB) medium (50ml), with 5 plugs (agar + fungi) for 3 days (under agitation) according to (Atoui et al., 2012), with modifications as follows: for approximately 200 mg of powder (ball or mortar grinding). 1 ml of extraction buffer (200 mM Tris – HCl, pH 7.5; 288 mM NaCl; 25 mM EDTA, pH8.0; 0.5% SDS) was added and vortexed vigorously until homogenized. Tubes were centrifuged at 12,000 rpm for 5 minutes (4°C). 750 µL were then transferred to a new tube, and 215 µL of 3M potassium acetate were added, mixed and incubated on ice for 30 min, centrifuged at 12,000 rpm for 15 min at 4°C. 700 µL of the supernatant were collected, transferred to a new tube and 500 µL of cold isopropanol (-20°C) were added. Tubes were centrifuged at 12,000 rpm for 15 min at 4° C. The supernatant was removed, and the pellet washed with 500 µL of cold 70% ethanol (-20°C) for 5 min (4°C). The tubes were vortexed, and centrifuged at 12,000 rpm for 5 min at 4° C. The supernatant was removed, the pellet was briefly dried, and resuspended in 200 μL of sterile milliQ H2O + 1 mg/ml RNase A (1/10 stock solution at 10 mg/ml). The sample was incubated for 1h at 37° C, and DNA concentration was done using NanoDrop. As control, 2 to 5 µL of each sample was run on 0.8% agarose gel.

*DNA sequencing:* Further *Trichoderma* identification was done by sending purified PCR product for sanger sequencing to the Eurofins platform (Germany, https://eurofinsgenomics.eu/en/custom-dna-sequencing/). This was done through partial sequencing of the transcribed spacer sites (ITS) (550 bp), the Calmodulin gene (CAL) (458 bp) and the Elongation Factor (EF) gene (350 bp). The oligonucleotides used for amplification and sequencing are: for the calmodulin (CAL-228F: GAGTTCAAGGAGGCCTTCTCCC/CAL-737R: CATCTTTCTGGCCATCATGG), for the elongation factor gene (EF1-728F: CATCGAGAAGTTCGAGAAGG/EF1-986R: TACTTGAAGGAACCCTTACC), and for the internal transcribed space (ITS1: TCCGTAGGTGAACCTGCGG/ITS4: TCCTCCGCTTATTGATATTGC). The amplification conditions were made as follows: 2 min at 95°C; 30 s at 95°C, 30 s at 60°C and 1 min at 72°C for 35 cycles. Each PCR reaction contained a total 50 µl consisting of 10 µl of 5x greenTaq buffer, 2 µl of 10 µM dNTP, 1,25 U of GoTaq^®^DNA Polymerase, 2 µl of each primer at 10 µM. The nucleotide sequences were compared with those deposited in GenBank using the BLAST program on the NCBI website as well as the similarity index. The sequences were aligned and constructed using the CLUSTALW software (https://npsa-prabi.ibcp.fr/cgi-bin/npsa_automat.pl?page=/NPSA/npsa_clustalwan.html).

The phylogenetic tree was constructed using the MEGA-X program (Kumar et al., 2018). The sequences used correspond to a concatemer of the CAL, EF and ITS sequences. (**Supplementary Table 1**) shows detailed supplementary information for the strains used in the phylogenetic analyses, with their corresponding geographic origin, substrate, voucher number, and GenBank accession numbers. Briefly, the evolutionary history was inferred by using the Maximum Likelihood method, and Tamura-Nei model (Tamura and Nei, 1993). The bootstrap consensus tree, inferred from 1000 pseudoreplicates (Felsenstein, 1985), is taken to represent the evolutionary history of the taxa analyzed (Felsenstein, 1985). Branches showing bootstrap values lower that 50% were collapsed. The percentage of replicate trees, in which the associated taxa clustered together in the bootstrap test (1000 replicates) were shown next to the branches (Felsenstein, 1985). Initial tree (s) for the heuristic search were obtained automatically by applying Neighbor-Join, and BioNJ algorithms, to a matrix of pairwise distances, and were estimated using the Tamura-Nei model, and then the topology, with superior log likelihood value was selected. This analysis involved 41 nucleotide sequences.

### Morphological characterization of the *Trichoderma* strains

The fungal strains were maintained on PDA with glucose at 26°C for 7 days. Microscopic visualization of *Trichoderma* conidiophores was done using fungi grown for 3 days on a PDA plate complemented with glucose (Glc). Briefly, the adhesive tape method was used in order to visualize the fungus. The fungus was grown for 3 days and a piece of adhesive tape was added over the fungus in order to stick them for visualization under the microscope.

### Endophytism tests

The endophytic capacity was tested using the Apogee spring wheat variety. The endophytism behavior of the 2 *Trichoderma* strains was examined using confocal microscopy. Two methods of inoculation were used and described below as the PDA inoculation method and the spore inoculation method. This experiment was repeated twice, and 5 samples were taken for each condition (with and without *Trichoderma*) for analysis.

For the PDA inoculation method, the seed sterilization protocol was modified from Jaber (2018). Seeds were soaked for 5 min with bleach (0.6% sodium hypochlorite) with stirring (150-200 rpm), then rinsed three times for 10 min with sterile milliQ water. They were then stratified for 3 to 5 days in the dark at 4°C, in distilled sterilized water. The inoculation was done on a PDA with Ampicillin (100 μg/ml) and Chloramphenicol (34 μg/ml) dish. Five wheat seeds were spread over a circle of 3 cm diameter and cultured at 26°C and in the dark. 24 hours later, a *Trichoderma* plug was added (7 days of culture on PDA) in the center of the petri dish and incubated at 26°C. Finally, roots were harvested after 5 days of culture.

For the spore inoculation method, the seed sterilization was carried out as mentioned above. The coating of the seeds with *Trichoderma* spores was then done as follows: the seeds and a spore solution coming from a 7-day-old *Trichoderma* colony on plate. Briefly, the spores were rubbed off using sterile distilled water and then were filtered in order to remove the germinated hyphae. This was followed by measuring the concentration using Thoma’s-cell slide to get a 10^7^ spores/ml final concentration. The solution was placed under shaking for 20-30 min, and then dried for a few minutes under the hood, before sowing. Seeds were then sown in glass tubes containing ½ MS (2.2g/L) supplemented with phytagel (2g/L).

*Root samples treatment for imaging*: After 5 days of incubation, roots were collected, gently cleaned from the excess of external fungal colonization, cut in 1.5/2.0 cm length, and washed with water or PBS 1X: 20 min. Root fragments were fixed overnight in a mixture of 95% Ethanol - 100% acetic acid (3: 1) (v: v). Samples were then stabilized for 1h in 1X PBS stabilizing solution.

Roots were included in 1X PBS with 3% agarose for 5 min (“Seaken LE Agarose for electrophoresis”) (0.6 g of agarose in 20 ml). For staining, cross-sections were immersed in the staining solution (20 µg/ml propidium iodide; 10µg/ml WGA-Alexa Fluor 488, 0.02% Tween 20 made up in 1X PBS) for 2 min, immersed in water for few seconds to remove the staining solution excess. Cross sections of wheat roots were visualized to check endophytism using Confocal microscope (Zeiss LSM880).

### Tests for wheat growth promotion

To test the effect of the *Trichoderma* strains on wheat root development, *Trichoderma* spores (10^7^ spores/10 ml) were incubated with wheat seeds in a tube containing 0.5X MS medium (Kapusi and Stoger, 2022) with phytagel without sugar. 10 days after germination, we measured the primary root growth. To evaluate wheat yield under controlled conditions, thirty-six 10-day-old plantlets, from seeds, pre-cultured on both, PDA (PDA approach), and phytagel ½ MS media (tube approach), were used. 32 plantlets from each treatment (with, and without *Trichoderma*) were transplanted to sterilized peatmoss soil (www.jiffygroup.com- Substrates for the professional horticulture) with perlite (3:1). All plants were kept in a growth chamber, under 16h: 8h light to dark period, with 265 μE m−2 s−1, at 22 °C, and 20 °C, day to night temperatures, and 60% relative humidity (RH). Regular irrigation was practiced, in parallel with fertilization practices at 14:12:32 N:P:K, plus 41 g Hortrilon micronutrients (B 0.5%, Cu 2.5%, Fe 5.0%, Mn 2.5%, Mo 0.5%, and Zn 0.6%) (https://www.fertil.fr/).

Wheat yield and yield components, including number of spikes/plants, number of grains/plants, grain weight/plant (g), whole plant dry weight (g), hundred grain weight (g), and harvesting index (HI%) were then measured.

### Trichoderma auxin production

Auxin production was measured using the Gusmiaty method (Gusmiaty et al., 2019) with some modifications in presence or absence of tryptophan.

Briefly, five plugs of each *Trichoderma* strain with, and without tryptophan were incubated in 0.5X MS liquid medium (2151,45 mg/L), 2-(N-morpholino) ethanesulfonic acid (MES) (0.25 g/L), glucose 2%, pH = 5.78 adjusted with 1M KOH (3 biological repeats).

To determine the auxin concentration (Indole-3-acetic acid or IAA in our case), Salkowski reagent, and IAA standard curve were done, according to (Gusmiaty et al., 2019). A standard IAA solution curve showing the relationship between the standard IAA solution (x), and its absorbance (y), was made, and allowed the quantification of IAA production by *Trichoderma* using the following equation: Y= a+ bX (a = Intersep, b = Slope (Regression Coefficient), Y = Absorbance, X = Concentration).

### Preparation of total protein extracts and SDS-PAGE

Total protein extracts were prepared from wheat grains grounded in liquid nitrogen using a mortar and pestle. From 50 mg of flour, total soluble proteins were extracted at room temperature in 960 μl thiourea/urea lysis buffer (Harder et al., 1999) containing 7 M urea, 2 M thiourea, 6 mM Tris-HCl, 4.2 mM Trizma^®^ base (Sigma-Aldrich, Lyon, France), 4% (w/v) CHAPS) supplemented with 162 μl of the protease inhibitor cocktail Complete Mini (Roche Diagnostics France, Meylan, France). Then, 22,5 μl of dithiothreitol (DTT, 1 M, Sigma-Aldrich), 4 μl of DNase I (Roche Diagnostics) and 10 μl of RNase A (Sigma-Aldrich) were added to the sample. For each sample, the protein extract was stirred for 30 min at 4**°**C and then centrifuged (35,000g, 15 min) at 4°C. The supernatant was submitted to a second clarifying centrifugation as above. The final supernatant corresponded to the total protein extract. Protein concentration in each extract was measured according to Bradford (1976). Bovine serum albumin was used as a standard. Separation of proteins was performed by sodium dodecyl sulphate polyacrylamide gel electrophoresis (SDS-PAGE) with a 4% (w/v) polyacrylamide stacking gel and a 12% (w/v) polyacrylamide resolving gel as described by Laemmli (1970). Electrophoresis was carried out at a constant current of 110 volts (10 mA) for 2 h using a Tris-glycine running buffer with of 0.2%. After electrophoresis, proteins were stained with GelCode^®^ Blue Stain Reagent (Thermo Scientific, Rockford, IL).

### *In vitro* growth antagonism

The capacity of the two *Trichoderma* strains to inhibit growth of another fungus was assessed against the plant pathogen *Fusarium graminearum*. Antagonism was tested by growth inhibition using 12 cm square plates. A plug of each fungus was placed in opposite corners on the plate and the growth inhibition was calculated by comparing the fungal growth (colony radius) from a plate inoculated with only one fungus. The growth medium was PDA.

### Statistical analysis:

Statistical analysis was conducted for the treatments, on a completely randomized design (CRD), and a factorial completely randomized design, with 32 replicates. The R software (R 3.3.4; R development core team, 2017) was used to analyze experimental data, calculate means, standard deviations, bar charts, and interaction, using the R package “ggplot2” (Wickham, 2009). Analysis of variance (ANOVA) was performed using the “lmerTest” R package (Kuznetsova et al., 2017).

## Results

### Characterization of two Lebanese *Trichoderma* strains isolated from wheat

In order to isolate *Trichoderma* fungi associated to wheat in Lebanon, 40 fungal isolates were isolated in total from three sampling sites. 21 were isolated from soil and 19 were isolated from plants. DNA was prepared from them and the ITS sequences amplified and sequenced. Based on the ITS sequences from these 40 colonies, 2 fungal isolates of the genus *Trichoderma* were identified (**Supplementary Table 2**). The two isolates were derived from site 3 (Doueir) and were named S3PA (isolated from stem base), and S3SB (isolated from soil). The other isolates included fungi from the genus: *Alternaria, Aspergillus, Boeremia, Cladorrhinum, Fusarium, Macrophomina, Microdochium, Mucor, Penicillium, Stromatinia*, and *Talaromyces* (**Supplementary Table 2**).

**Figure 1:**
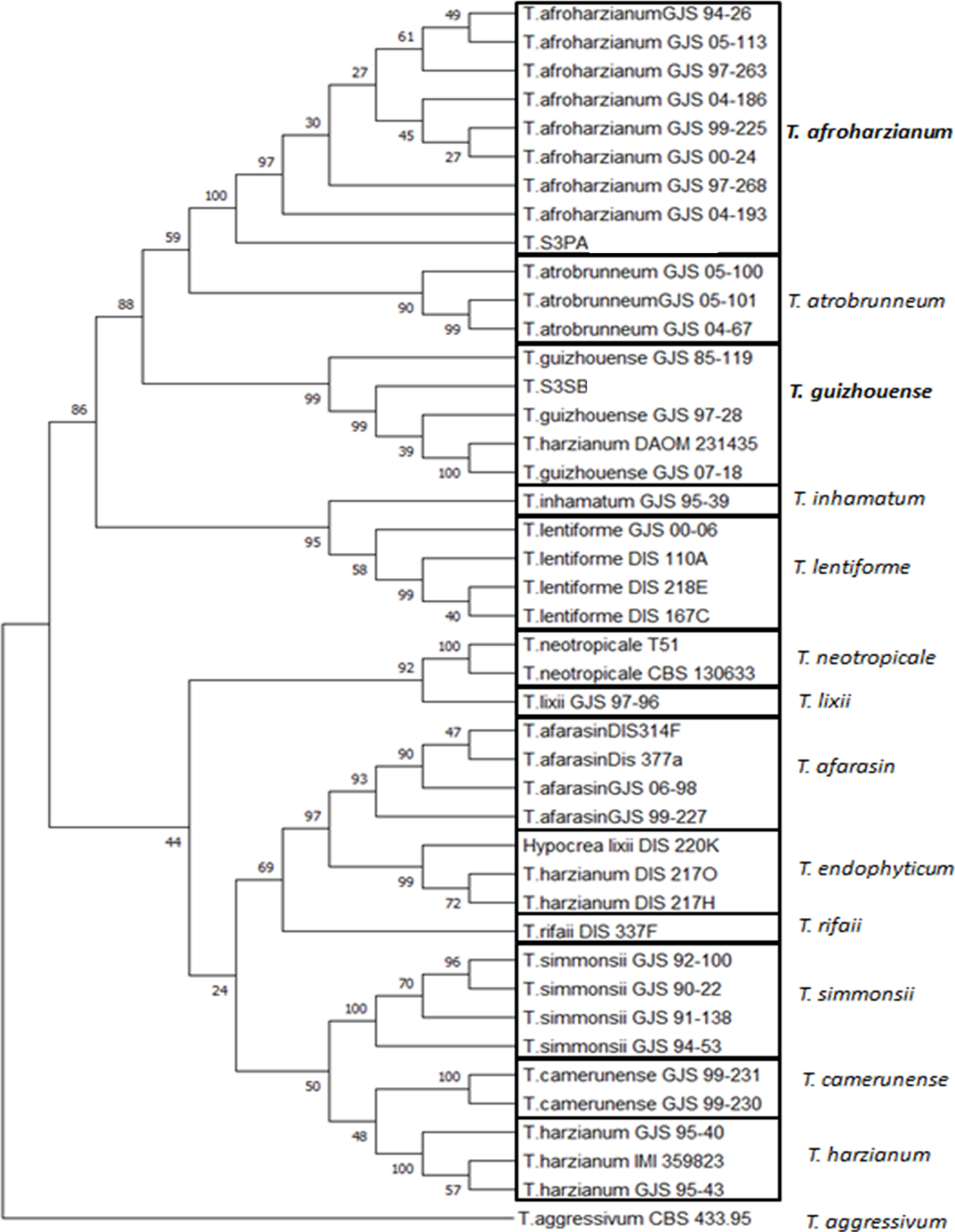
Phylogenetic tree of CAL-EF-ITS branch includes only species in the *T. harzianum* complex. Two species are identified from Lebanon and are placed in this tree: *T. afroharzianum* (T. S3PA) and *T. guizhouense* (T. S3SB). Strains used in this phylogenetic analysis are described in **Supplementary Table 1** with their corresponding geographic origin, substrate, voucher number and GenBank accession numbers. *T. agressivum* is used as an outgroup in this analysis.

A more precise molecular description of these two *Trichoderma* fungi was done by characterizing the partial sequences of their Elongation factor (EF-1) and Calmodulin (Cal) gene sequences. The three partial sequences (ITS, EF and Cal) were then concatemerized (550, 350, and 489 bp, respectively) and compared to the same concatemerized sequences of 39 strains representing 14 *Trichoderma* species (**Supplementary Table 1**) from the public database and representing the *Trichoderma* diversity. The result of this analysis (**Figure 1**) showed that the two isolates probably belong to the two species *T. afroharzianum* and *T. guizhouense* belonging to the *Harzianum* species complex.

### Morphological characterization of the two Lebanese *Trichoderma* strains

**Figure 2:**
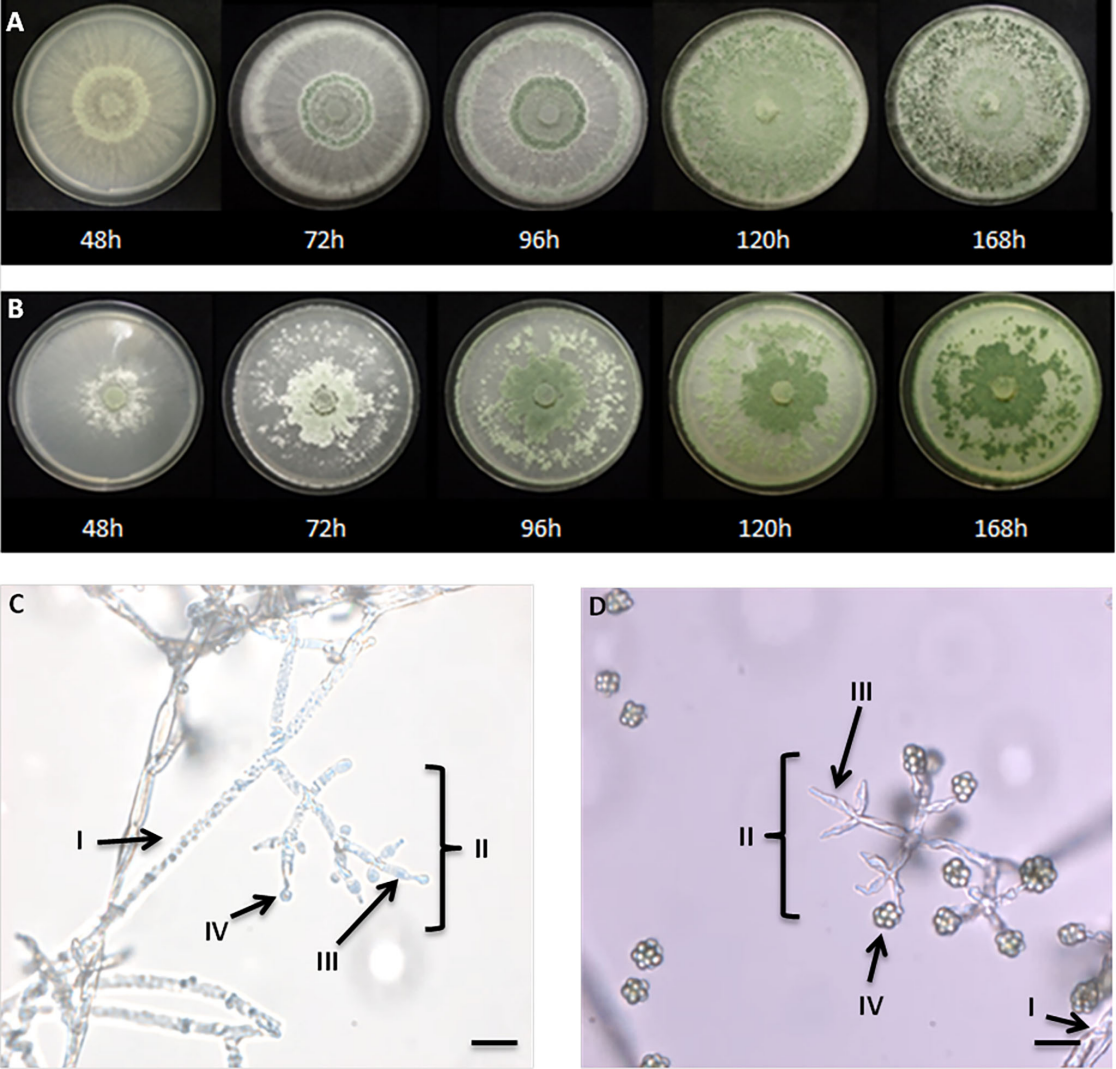
Growth of *T. guizhouense* **(A)** and *T. afroharzianum* **(B)** for one week on PDA supplemented with glucose. The age of the culture is indicated below the picture in hours. Microscopic visualization of conidiophores of *T. guizhouense* **(C)** *T. afroharzianum* **(D)** (x1000). (I) hyphae, (II) conidiophores, (III) Phialides and (IV) conidia (the scale bar corresponds to 10μm).

After 48h at 26°C on PDA, the *T. guizhouense* formed an abundant aerial mycelium, cottony and radiating a white color. At 72 h, the formation of a greenish ring was observed, which increased by time of incubation, and diffused in the media, until covering almost the whole petri dish at 168 h. The conidia were visualized from 48h in broad concentric bands (green), in the aerial hyphae (**Figure 2A**). After 72h of growth on PDA (26°C), *T. afroharzianum* produced an abundant aerial mycelium, cottony, radiating a white color (**Figure 2B**). A dense hyphae growth in the petri dish center was observed after transplantation of *Trichoderma*, while 96 h later, the formation of a greenish ring was observed, which increased with the time of incubation, and diffused in the media, until covering almost the whole petri dish at 168 h.

We then morphologically characterized the two newly isolated strains. After 3 days, pyramidal conidiophores were repeatedly branched, irregularly arranged in whorls, and showed clusters of divergent, usually unevenly folded, barrel shaped to nearly sub-globose phialides. The ellipsoidal to globose conidia tend to be greenish to light turquoise, sometimes hyaline to cluster in aggregates at the terminal of the phialides (Zhu and Zhuang, 2015). Conidiophores of *T. guizhouense*, showed opposite branches. The main axis and each branch terminated at a cruciate whorl of 3 ampulliform phialides (**Figure 2C**). Conidia, appearing within 48–72 h, were typically abundant, and disposed in one, or three concentric rings, around the point of inoculation. Green pigment sometimes diffused in the medium (**Figure 2B**). After 3 days of incubation on PDA, the pyramidal conidiophores of *T. afroharzianum* showed opposite branches. The main axis, and each branch terminated at a cruciate whorl of 3 ampulliform phialides green to dark green when aging (**Figure 2D**).

The two isolated strains thus grow rapidly on PDA medium with phenotypic characteristics of other *Trichoderma* stains (Chaverri et al., 2015).

### The Lebanese *Trichoderma* strains are wheat root endophytes

**Figure 3:**
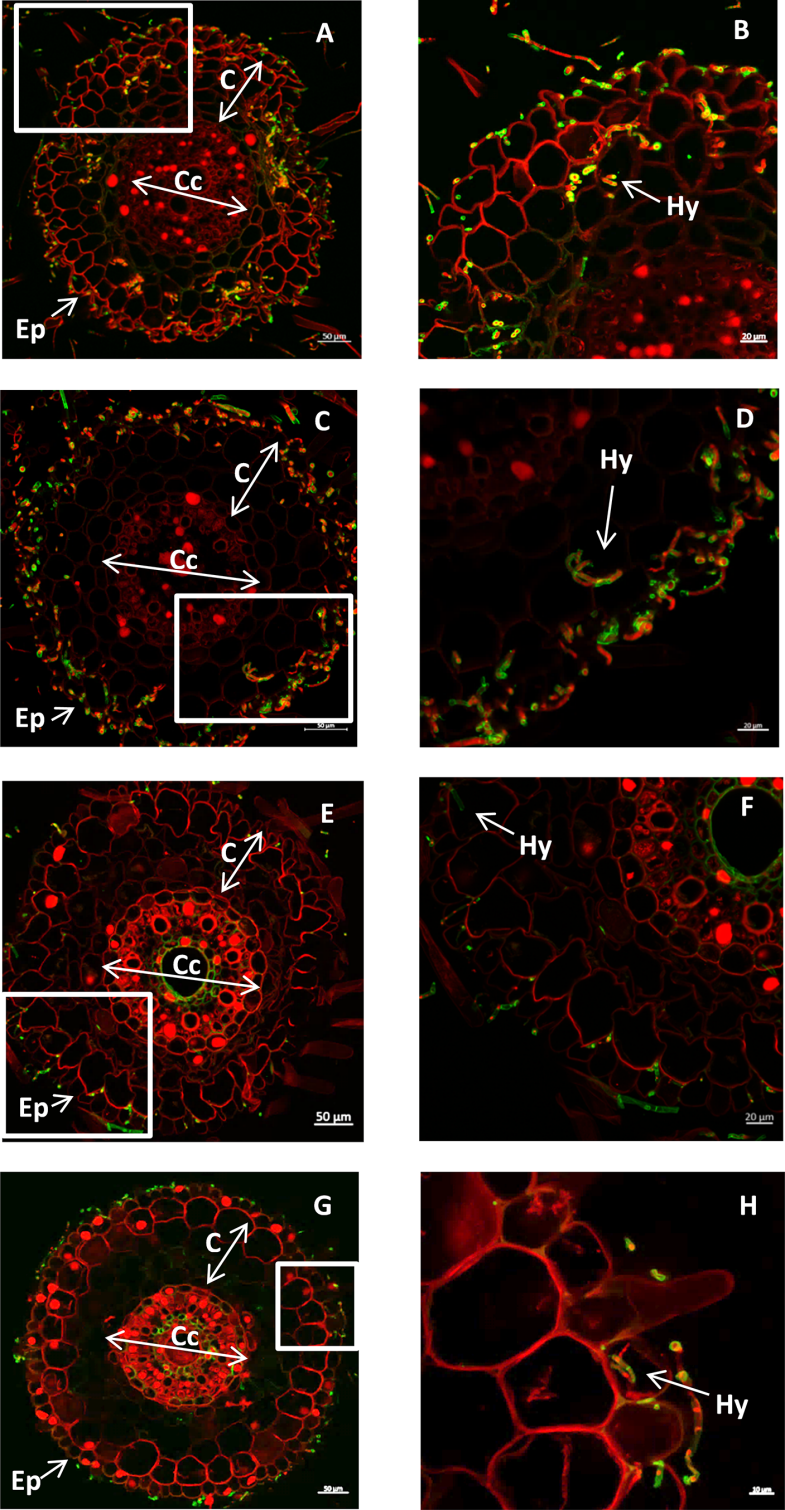
**(A–D)** cross section of wheat root seedlings inoculated with *T. guizhouense* **(A, B)** or *T. afroharzianum* **(C, D)** using the PDA inoculation method. E-H: cross section of wheat root seedlings inoculated with *T. guizhouense* **(E, F)**, *with T. afroharzianum* **(G, H)** using the spore inoculation method. (Ep) epidermis, **(C)** cortex (Cc) central cylinder, (Hy) fungal hyphae. The fungal hyphae are stained green using WGA-Alexa Fluor 488, and the root cell walls are stained red using propidium iodide, (BDFH correspond to the boxes of ACEG respectively).

*Trichoderma* fungi were previously described as endophytes (Tseng et al., 2020). In order to know if the two Lebanese strains isolated from wheat behave also as endophytes, two modes of inoculation were tested, either by inoculating the seeds by co-cultivation on PDA medium (PDA inoculation method), or by spore coating of the seeds and germination of the seeds *in vitro* (spore inoculation method, see Material and Method section). The difference between the two methods is that in the PDA method the seeds were inoculated with undetermined high concentrations of spores and fungi whereas with the spore coating method the spore number was controlled. In both cases the root sections of the plants were stained with propidium iodide and WGA-Alexa Fluor 488, staining the fungus in green. These sections were observed using confocal microscopy. Using both inoculation methods, fungal hyphae were observed inside the root for the two *Trichoderma* strains (**Figure 3**). The infection level observed using the PDA inoculation method seemed to be higher than the spore inoculation method that had a lower concentration of the spore solution. In addition, *T. guizhouense* was isolated from soil infected rhizodermis and cortex cells using the first methods (**Figures 3A, B**) and mostly rhizodermis using spore coating (**Figures 3E, F**). The *T. afroharzianum* strain appears less efficient in root colonization, invading mostly the rhizodermis cell layer using the PDA inoculation (**Figures 3C, D**) and colonizing only the root surface using spore coating (**Figures 3G, H**). Using leaves of 4-weeks-old plants, we were unable to reisolate the inoculated fungi, suggesting that these *Trichoderma* strains are not able to colonize the aerial part of the plant.

All together this experiment shows that the two strains can behave as fungal wheat root endophytes but that *T. guizhouense* can invade root tissue more efficiently than *T. afroharzianum* and that the inoculation method can influence the level of colonization.

### The two *Trichoderma* strains promote wheat root growth and wheat aerial development and produce auxin

*Trichoderma* strains have been often used as plant growth promoting microorganisms (Tseng et al., 2020). In order to know if the two strains studied here can also behave as plant growth promoting fungi (PGPF), we tested their growth promotion effect on 36 young seedlings *in vitro*, using the spore coating method and ½ MS medium without sucrose.

**Figure 4:**
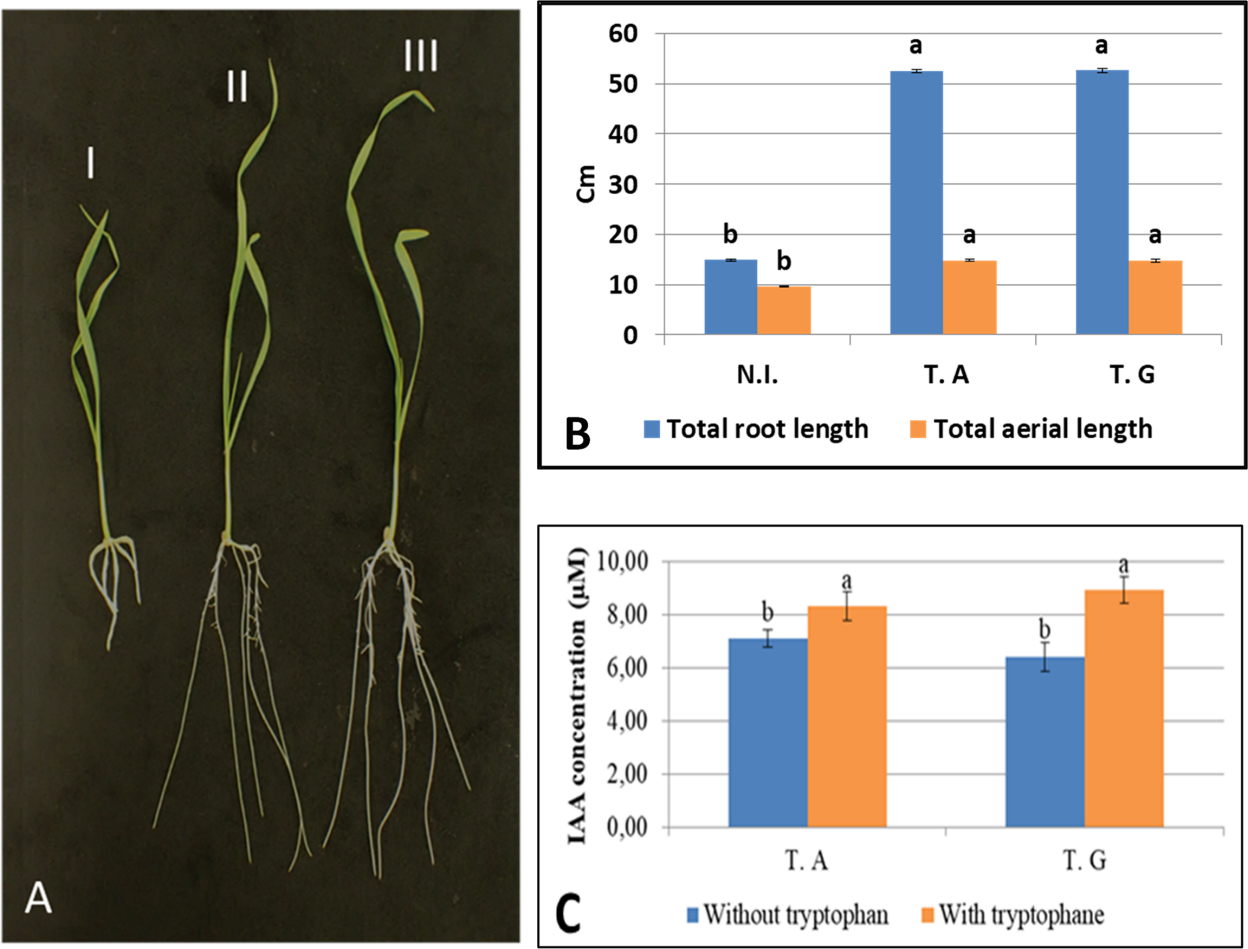
Growth of roots and aerial parts after 10 days of culture, with and without *Trichoderma* **(A)**; I) not inoculated, II) a representative seedling inoculated with *T. guizhouense*, III) a representative seedling inoculated with *T. afroharzianum*, **(B)** Total root, and aerial length (cm) of non-inoculated (N.I.) or inoculated wheat plants with *T. afroharzianum* (T.A) and *T. guizhouense* (T.G), respectively, **(C)** IAA production capability of the two *Trichoderma* isolates (T.G and T.A) with and without tryptophan. Values in the same graph having different letters are significantly different, at P ≤ 0.01 (ANOVA test).

This experiment shows that the presence of the two *Trichoderma* strain resulted in a significant increase of the primary, and secondary root growth, as well as the aerial part of the plant as early as 10 days post germination (**Figure 4A** and **Supplementary Figure 1**). Total root, and aerial length (cm) of inoculated wheat plants, with and without *T. guizhouense* and *T. afroharzianum* respectively, is shown in **Figure 4B**. The results indicated that plants incubated with either *T. guizhouense* or *T. afroharzianum* had increased root growth and aerial development as compared to non-inoculated plants. Root length was increased by 3.5x and 3.5x for plants inoculated by *T. afroharzianum T. guizhouense* respectively. For the aerial part we observed a 1.5x increase in length with both fungi. In this experiment, the inoculation by the *Trichoderma* strains enhanced principal root length (2.5x), total root length (3.4x), and aerial part lengths (1.5x) as well as total aerial plant weight (**Figure 4B** and **Supplementary Figures 1A, B**).

Total root, and aerial mass of wheat plants, with and without *T. guizhouense* and *T. afroharzianum* respectively are shown in **Supplementary Figure 1B**. The inoculation with *T. afroharzianum* resulted in an increase of 2.6x for the total root mass and 1.9x, for total aerial mass, while the treatment with *T. guizhouense* resulted in an increase of 2.7x for total root mass and 2x, for the aerial mass.

We further measured the principal root length of inoculated wheat plants over a 10 days period (kinetics), with and without *T. guizhouense* or *T. afroharzianum*. The root growth promoting effect of the two strains was already detectable 2 days after sawing and continued to increase over the 10 days period (**Supplementary Figure 1B**).

The increased root growth observed in presence of the two strains might result from fungal auxin production. We thus measured auxin production by the two *Trichoderma* strains in culture, in presence and absence of tryptophan, an auxin precursor. This experiment (**Figure 4C**) shows that the two strains can produce auxin in culture and that addition of tryptophan enhances IAA production by 1.2x, and 1.4x, for *T. guizhouense* and *T. afroharzianum* respectively.

These experiments show that wheat inoculation by the *Trichoderma* Lebanese strains enhances root growth and this might result from auxin production by the fungi.

### Inoculation by the *Trichoderma* Lebanese strains enhances wheat yield

**Figure 5:**
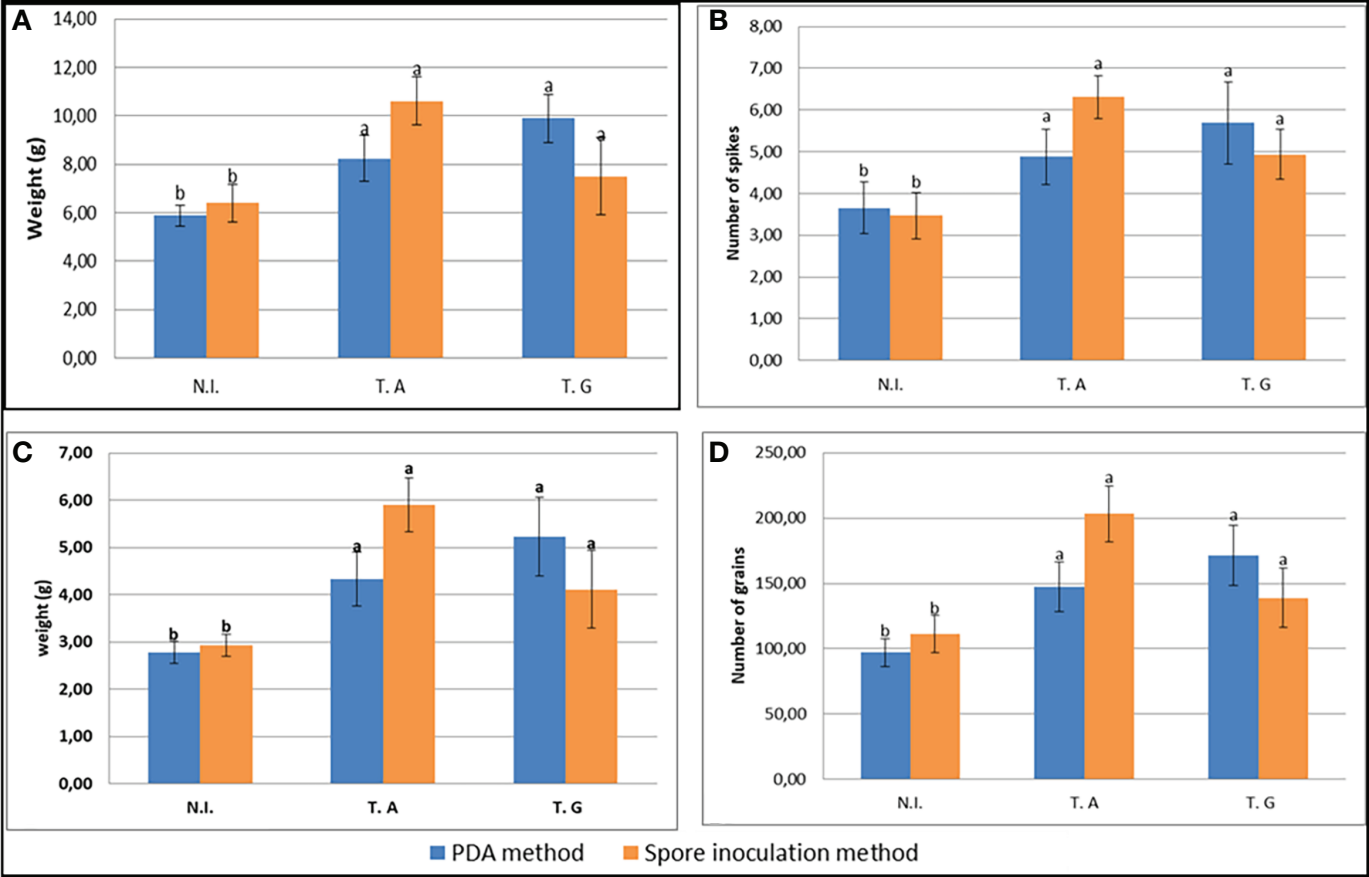
Yield parameters: **(A)** Total dry plant weight (g)/plant; **(B)** Number of spikes/plant; **(C)** Total grains weight (g)/plant; **(D)** Number of grains/plant, as influenced by PDA and spore inoculation methods. N.I.: T.A: wheat plants inoculated with *T. afroharzianum*; T.G: wheat plants inoculated with *T. guizhouense* Values in the same graph having different letters are significantly different, at P ≤ 0.01 (ANOVA test). 32 plants were analyzed per sample.

As shown above, *Trichoderma* inoculation by the two *Trichoderma* strains enhanced wheat seedling growth. We thus tested their effect on the complete development of the wheat plants grown in soil. The two inoculation methods described above were used before plant transplantation in pots but no significant difference could be observed as a result of the inoculation method. Plants were grown to maturity and the different wheat yield components were measured (**Figure 5**; **Supplementary Figure 2**). This analysis showed that the plant weight (**Figure 5A**), the number of spikes per plant (**Figure 5B**), the total grain weight per plant (**Figure 5C**) and the grain number per plant (**Figure 5D**) increased following inoculation by the two fungi. In contrast the 100 grains weight remained unchanged (**Supplementary Figure 2A**). Altogether, yield harvest index (HI%) increased around 1.1x (PDA inoculation method) and 1.2x (spore inoculation method) by the fungal inoculations (**Supplementary Figure 2B**).

In order to know whether fungal inoculation could change seed protein quantity as well as the seed protein pattern of the grains we analyzed them in plants inoculated by the two strains and following the two inoculation methods. As shown in **Supplementary Figure 2C** neither the protein contents nor the protein profiles were modified in the wheat grains by the *Trichoderma* inoculation.

Thus, inoculation of plants, independently of the method used increased wheat yield including number of spikes/plants, number of grains/plants, grain weight/plant, whole plant dry weight, and harvesting index (HI%), but did not modify the seed protein content.

### The two Trichoderma strains inhibit Fusarium growth *in vitro*

**Figure 6:**
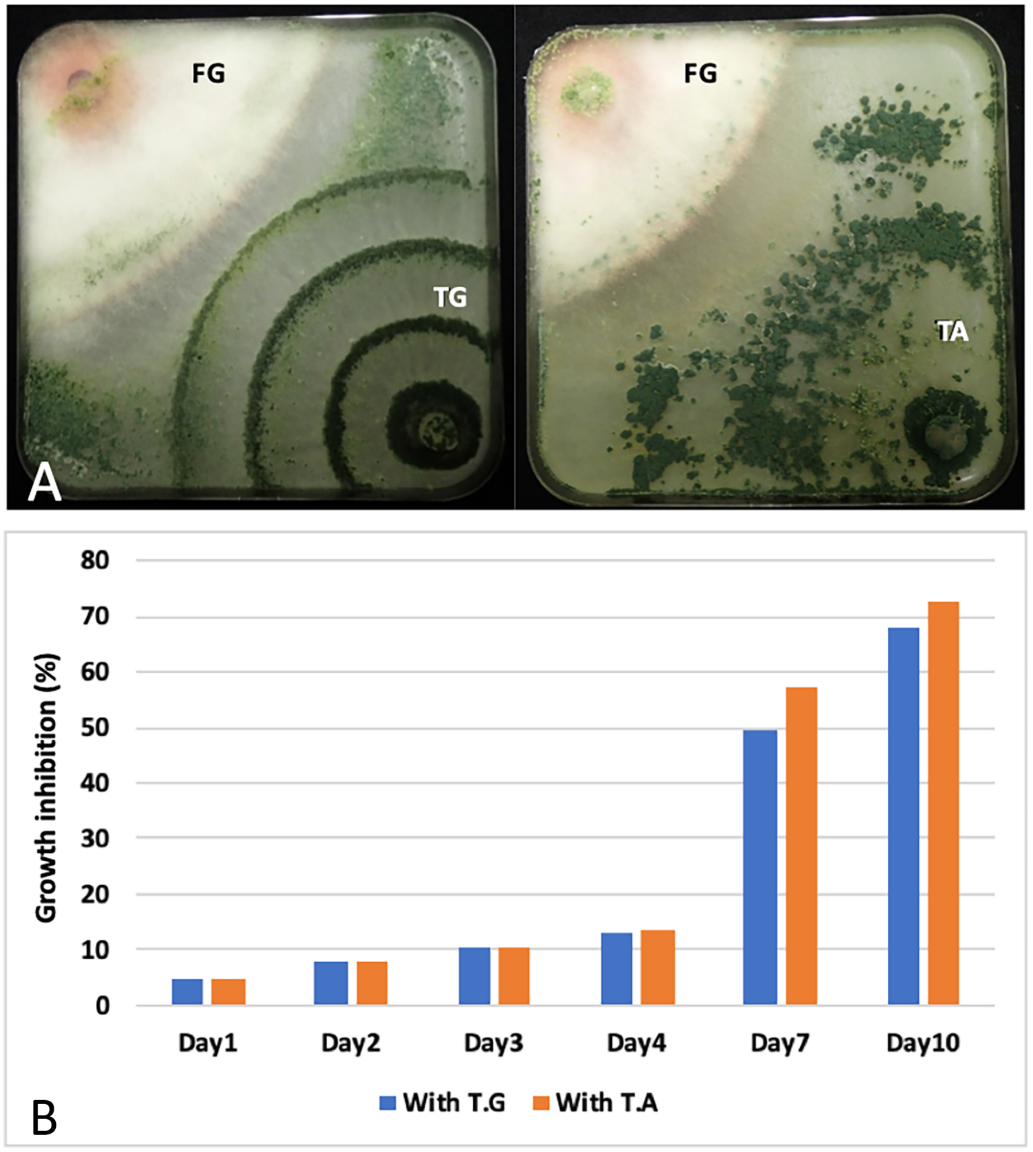
Growth antagonism: **(A)** Antagonistic effect of *T. guizhouense* (TG) and *T. afroharzianum* (TA) against the plant pathogenic fungi, *Fusarium graminearum* (FG), 10 days post inoculation. **(B)** Kinetics of mycelial growth inhibition of *F. graminearum* in the presence of *T. guizhouense* and *T. afroharzianum*. The inhibition is expressed as the percentage (%) of growth inhibition compared to plates inoculated with only one fungus. Fungi were inoculated in opposite corners of the square petri dishes.

In order to know if the two Lebanese strains also possess antifungal activity, we tested their growth inhibition activity against *F. graminearum in vitro*. To do this, a plug of a fungal colony was place in one corner of a square petri dish plate (12cm) containing PDA. To test the inhibition, the two fungi were inoculated on opposite corners (**Figure 6A**). Doing this, the contact between the two fungi was observed at day 7 (contact day). Beyond this period and after 10 days, *T. guizhouense* and *T. afroharzianum* invades the colonies of *F. graminearum* and even sporulates on them (**6A**). The percentage of growth inhibition was calculated using plates only inoculated by *F. graminearum* and measuring the radius of the colony each day. This experiment shows that *T. guizhouense* and *T. afroharzianum* inhibit *F. graminearum* growth even before the contact between the fungal colonies, and that the % of inhibition of mycelial growth reached 50% and 58% respectively, after 7 days (contact day; **Figure 6B**).

## Discussion

Plants interact with many microorganisms in nature and it is believed that a better understanding of these interactions can help develop sustainable agriculture less dependent on pesticides and polluting fertilizers. Among interacting microorganisms, some are deleterious (pathogens) but others are beneficial (Singh et al., 2018; Kakabouki et al., 2021). The fungus *Trichoderma* is widely studied as a beneficial microorganism, promoting plant growth but also protecting plants against pathogens through a variety of mechanisms (Stewart and Hill, 2014; Mukhopadhyay and Kumar, 2020; Sood et al., 2020; Shahriar et al., 2022). Among crops, wheat is one of the major staple foods around the world. Previous studies have shown that it can benefit from interaction with *Trichoderma* fungi for growth promotion or pathogen protection (TariqJaveed et al., 2021).

In this study we have isolated two *Trichoderma* strains from Lebanese soils, characterized them, and evaluated their growth promotion effect on wheat. These two strains were isolated amongst 40 fungal isolates from soil or plant tissues. The storage and/or isolation methods can explain the low frequency (5%) of *Trichoderma* strains isolated in this experiment. Molecular and taxonomic characterization showed that the two strains are *T. guizhouense*, and *T. afroharzianum* isolates belonging to the Harzianum complex (Chaverri et al., 2015; Stummer et al., 2020). The morphology of the two strains is in agreement with *Trichoderma* morphology (Zhu and Zhuang, 2015). In addition, these two isolates were also able to colonize the root rhizoderm, and external cortex, while markedly enhancing seedling root growth. In contrast, our study suggested that the two Lebanese isolates lack the ability to colonize wheat aerial tissues under the conditions used. Growth conditions might influence colonization as the *T. afroharzanium* strain was isolated from the base of the aerial part of a field grown plant.

The influence of the two *Trichoderma* strains inoculation on wheat root development suggested that they may produce auxin (Nieto-Jacobo et al., 2017). IAA is the main auxin promoting root system development, and able to modify root architecture in the *Trichoderma* plant interaction (Illescas et al., 2021). Additional *Trichoderma* metabolites, and proteins (effectors) are also known to significantly modulate IAA production in plants, resulting in root hair growth and enhanced root mass development (Illescas et al., 2021). IAA biosynthesis in fungi is tryptophan-dependent, and supplying this amino acid to the medium, increases IAA production (Nieto-Jacobo et al., 2017). We indeed could enhance IAA production in culture of *T. afroharzianum*, and *T. guizhouense* by adding tryptophan to the medium. These results are in agreement with previous studies showing that auxin-generating *Trichoderma* strains interact with wheat and other plants (Pelagio-Flores et al., 2017; Illescas et al., 2021).

Approaches of inoculating *Trichoderma* to promote plant growth varied between seed/seedling treatments, soil processing, and foliar spray (Bhandari et al., 2021). Recent studies caried out on model plants like *Arabidopsis thaliana* inoculated by *T. atroviride, and T. guizhouense* strains showed that *Trichoderma* volatiles increased endogenous sugar contents in shoots, roots and root exudates, and could enhance *Arabidopsis* root development, root architecture, and promote the symbiosis efficiency. These effects were suggested to be related to auxin transport and signaling (Esparza-Reynoso et al., 2021; Li et al., 2022). *Trichoderma* fungi can also stimulate root development and modify plant metabolism, by releasing numerous secondary compounds (Kakabouki et al., 2021). For example, harzianic acid is one of *T. harzianum* metabolites that is responsible for promoting plant growth (Vinale et al., 2014; Xie et al., 2021) and acting as biocontrol agent. Further studies will indicate which mechanisms are responsible for the wheat growth promotion by our two strains.

In this work we tested the ability of *T. afroharzianum* and *T. guizhouense* to enhance wheat growth (plant growth promotion) using two seed inoculation methods: 1) PDA mediated inoculation, and 2) spore inoculation. Our results showed a notable increased in seedling development during the first 10 days following seed inoculation by the *T. afroharzianum*, and *T. guizhouense* fungi (**Figures 4A, B**). This result is in agreement with previous studies (Kucuk, 2014), which showed that root and shoot growth of wheat could be improved by *T. harzianum*, isolated from Turkey.

Remarkably, the wheat seedling growth was greatly increased when plants were inoculated by the two Lebanese strains, indicating that the interaction can be established very early during wheat development. Similar effects were observed when inoculating seedlings with other *Trichoderma* spp. (Mahato, et al., 2018; Oliveira et al., 2018; Anjum et al., 2020; Kthiri et al., 2020). These studies indicated that many growth or yield parameters like the percentage of germination, root length, shoot length, total length, fresh root mass, fresh shoot mass, total fresh mass, dry root mass, dry shoot mass, total biomass, root mass ratio, shoot mass ratio and aerial part/root system ratio, were augmented. Similarly, *T. yunnanense*, and *T. afroharzianum* isolates produced IAA under salt stress (200 mM), and could stimulate photosynthesis, water utilization, and growth of wheat in saline conditions (Zhang et al., 2019; Oljira et al., 2020).

Our study is also in agreement with previous studies (Colla et al., 2015; Silletti et al., 2021), where seed coating encouraged seed germination, and the quality of yield and grain, including protein and mineral composition in durum wheat. Xue et al. (2017) inoculated six different *Trichoderma* strains on wheat seedlings and found that the six strains had the ability of increasing plant dry weight, and total yield, over a three-year experiment. This is in line with our data showing that treatment with the two Lebanese *Trichoderma* strains could significantly affect plant yield. The HI% indicated an increased yield, as a response to treatments with both *T. guizhouense* and *T. afroharzianum* strains, but *T. afroharzianum* showed higher impact on yield and its components. The fungal growth promotion effects are not completely described but it is known that *Trichoderma* can stimulate plant food scanning by enhancing root elongation in order to invade more, either shallow surface, or even deeper underneath areas, seeking for nutrients uptake. This capacity probably essentially relies on auxin production (Zin and Badaluddin, 2020). In agreement with this, in our study *T. afroharzianum* and *T. guizhouense* isolated strains strongly promoted primary and secondary root elongation.

Protein content of grains is an important agronomic character for wheat (Illescas et al., 2022). Protein analysis of the grains revealed that in our experiments the inoculation by *Trichoderma* spp. did not modify their protein nature and content. This shows that *Trichoderma* can promote wheat growth and enhance grain production without changing the grain protein composition. This observation might be correlated with the other part of our study, indicating that the weight of a hundred grains is not changed despite yield increase.

The growth inhibition against *F. graminearum in vitro* indicates that like other *Trichoderma* strains, the two Lebanese strains can probably be used as biocontrol agents. Further experiments will be needed to show that these biological control activities are relevant in field conditions.

In conclusion, in this study, two *Trichoderma* strains were isolated from wheat plants and wheat rhizospheric soil in Lebanon. These fungi were identified as *T. guizhouense* and *T. afroharzianum* and behaves as root endophytes. In addition, they promote wheat seedling growth and yield.

## Data availability statement

The datasets presented in this study can be found in online repositories. The names of the repository/repositories and accession number(s) can be found in the article/**Supplementary Material**.

## Author contributions

NM, PR, MD, FA-S and NB conceived and designed the experiments. NM, CM, PR, MD, GA, AP, BC and LR conducted the experiments and analyses. PR, NM, GA, and FA drafted the manuscript. All authors have read and approved the final version of the manuscript.

## Funding

This work was funded by the ‘AUF-CNRSL-UL’, Lebanon Francophonie University Agency (AUF), the National Council for Scientific Research in Lebanon (CNRS-L) and the Lebanese University (UL). This work was also supported by the CNRS and has benefited from the support of the LabEx Saclay Plant Sciences (ANR-10-LABX-0040-SPS, LabEx SPS and ANR-17-EUR-0007, EUR SPS-GSR) which is managed by the French National Research Agency under the program ‘Investissements d’avenir’ (ANR-11-IDEX-0003-02) and the Plant2Pro, Carnot Institute project TrichoKissCool.

## Acknowledgments

The authors would like to thank ICARDA and LARI for supporting this research by providing samples and materials. Also thanks to Dr Muhsin Parson for careful reading and editing of the manuscript.

## Conflict of interest

The authors declare that the research was conducted in the absence of any commercial or financial relationships that could be construed as a potential conflict of interest.

## Publisher’s note

All claims expressed in this article are solely those of the authors and do not necessarily represent those of their affiliated organizations, or those of the publisher, the editors and the reviewers. Any product that may be evaluated in this article, or claim that may be made by its manufacturer, is not guaranteed or endorsed by the publisher.
