# Supplementary material for: Identification of beneficial Lebanese Trichoderma spp. wheat endophytes
Source: Front Plant Sci. 2022 Dec 2;13:1017890. doi: 10.3389/fpls.2022.1017890 (PMC9755858; doi:10.3389/fpls.2022.1017890)
Supplement: Supplementary file 1 [file DataSheet_1.pdf]

frontiers

Supplementary Material

1 Supplementary Tables

SUPPLEMENTARY TABLE I. Strains used in the phylogenetic analyses, with their corresponding geographic origin, substrate, voucher number and GenBank accession numbers

				Genbank accession Nos.		
**Species**	**Voucher/culture Nos.**	**Geographic origin**	**Substrate**	***CAL***	**ITS**	***EF1***
T. afarasin	CBS 130742<br>= Dis 314f	Cameroon	*Cola altissima* trunk endophyte	FJ442312	FJ442259	FJ463400
T. afarasin	Dis 377a	Cameroon	*Cola* sp. trunk endophyte	KP115277	FJ442665	FJ463322
T. afarasin	CBS 130501<br>= G.J.S. 06-98	Cameroon	Soil	FJ442353	FJ442630	FJ463327
T. afarasin*	CBS 130755<br>= IMI 393967<br>= G.J.S. 99-227	Cameroon	Soil	FJ442388	AY027784	AF348093

T. afroharzianum	G. Harman 1295-22<br>= ATCC 29847<br>= T22<br>= GJS 94-26	Colombia and New York[2]	G. Harman, patented biocontrol strain	AF469190	AF469188	AF469194
T. afroharzianum	G.J.S. 00-24	Mexico	Soil	AF442880	AF443922	AF443940
T. afroharzianum*	CBS 124620<br>= G.J.S. 04-186	Peru	On basidioma of *Moniliophthora roreri* on fruit of *Theobroma*	FJ442370	FJ442265	FJ463301
T. afroharzianum	CBS 124614<br>= G.J.S. 04-193	Peru	On basidioma of *Moniliophthora roreri* on fruit of *Theobroma*	FJ442372	FJ442233	FJ463298
T. afroharzianum	G.J.S. 05-113	Italy	Wheat seed	FJ442371	FJ442235	FJ463378
T. afroharzianum	G.J.S. 97-263	Japan	Soil	AF442877	AF194010	AF348091

T. afroharzianum	G.J.S. 97-268	Japan	Soil	AF442878	AF194015	AF348105
T. afroharzianum	IMI 393972 = G.J.S. 99-225	Cameroon	Soil	AF442882	AY027781	AF348106
T. aggressivum	CBS 433.95	Northern Ireland	Mushroom compost	FJ442279	FJ442605	AF348097
T. atrobrunneum	CBS 130440 = G.J.S. 04-67	Italy	Soil	FJ442329	FJ442273	FJ463360
T. atrobrunneum	G.J.S. 05-100	Italy	*Castanea sativa* twig	FJ442364	FJ442234	FJ463299
T. atrobrunneum	G.J.S. 05-101	Italy	Soil	FJ442331	FJ442677	FJ463392
T. camerunense*	CBS 137272 = G.J.S. 99-230	Cameroon	Soil	AF442875	AY027780	AF348107
T. camerunense	G.J.S. 99-231	Cameroon	Soil	AF442874	AY027783	AF348108
T. endophyticum	CBS 130730 = Dis 217h	Ecuador	*Theobroma gileri* trunk endophyte	FJ442293	FJ442242	FJ463314

T. endophyticum	Dis 217o	Ecuador	*Theobroma gileri* trunk endophyte	FJ442294	FJ442241	FJ463323
T. endophyticum	Dis 220k	Ecuador	*Theobroma gileri* trunk endophyte	FJ442299	FJ442270	FJ463328
T. guizhouense	DAOM 231435	Rwanda	Soil	FJ577721	EF191296	EF191321
T. guizhouense	G.J.S. 07-18	Ghana	Soil	FJ442355	FJ442641	FJ463390
T. guizhouense	ATCC 90179 = IMI 374787 = G.J.S. 85-119	Indonesia	Dead wood	AF442881	AF443923	AF443941
T. guizhouense	NBRC 30608 = IFO 30608 = G.J.S. 97-28	Japan	Dead branch	FJ442379	DQ018116	AY937440
T. harzianum*	CBS 226.95 = G.J.S 95-43	U.K.	Soil	AF442864	AJ222720	AF348101
T. harzianum	CBS 227.95 = G.J.S 95-40	U.K.	Soil	AF442866	AJ222721	AF348100

T. harzianum	IMI 359823	Northern Ireland	Mushroom compost	AF442865	X73690	AF348092
T. inhamatum*	CBS 273.78<br>= IMI 287526<br>= G.J.S. 95-39	Colombia	Soil	AF442891	FJ442680	AF348099
T. lentiforme	CBS 130726<br>= Dis 110a	Ecuador	*Theobroma cacao* trunk endophyte	FJ442287	FJ442681	FJ851872
T. lentiforme	Dis 167c	Brazil	*Theobroma cacao* trunk endophyte	FJ442365	FJ442269	FJ463309
T. lentiforme	Dis 218e	Ecuador	*Theobroma gileri* trunk endophyte	FJ442296	FJ442220	FJ463310
T. lixii*	CBS 110080<br>= ATCC MYA-2478<br>= G.J.S. 97-96<br>= BPI 745654	Thailand	Decayed *Ganoderma* basidiocarp	AF442872	AF443920	AF443938

T. neotropicale*	G.J.S. 11-185<br>= CBS 130633<br>LA11	Peru	*Hevea guianensis* trunk endophyte	KP115279	HQ022407	HQ022771
T. neotropicale	G.J.S. 11-187<br>= T51	Peru	*Hevea brasiliensis* trunk endophyte	KP115280	FJ884180	FJ967825
T. rifaii	CBS 130745<br>= Dis 337f	Panama	*Theobroma cacao* trunk endophyte	FJ442315	FJ442621	FJ463321
T. simmonsii	CBS 123799<br>= IMI 393966<br>= G.J.S. 90-22	USA, Wisconsin	Decorticated wood	AF442867	AF443915	AF443933
T. simmonsii*	CBS 130431<br>= G.J.S. 91-138	USA, Maryland	Decaying bark	AF442869	AF443917	AF443935
T. simmonsii	CBS 546.92<br>= ATCC MYA-2453<br>= G.J.S. 92-100	USA, Alabama	Decorticated wood	AF442871	AF443919	AF443937

T. simmonsii	CBS 130432<br>= G.J.S. 94-53<br>BPI 749348	USA, Illinois	Decorticated wood	AF442868	AF443916	AF443934

* Indicates a type culture.

** Indicates an epitype culture.

[1] CBS = CBS Fungal Biodiversity Centre culture collection, the Netherlands; DAOM. = Agriculture and Agri-Food Canada National Mycological Culture Collection; IMI. = CABI culture collection, UK; ATCC. = American Type Culture Collection, Manassas, Virginia, USA; BPI = US National Fungus Collection; WU = Herbarium WU, Institute of Botany, University of Vienna, Austria; G.J.S. = G. J. Samuels; P.C. = P. Chaverri; Hypo, C.P.K., S and WJ = W. Jaklitsch collection numbers; Dis = H.C. Evans endophyte cultures; LA, CM, PP, T = P. Chaverri endophyte cultures. Where a plant name alone is given, the substrate is twigs or branches and fungi growing on them.

[2] T22 is product of fusion of protoplasts of two cultures: ATCC 60850, which was isolated from soil in Colombia, and ATCC 20707, which was isolated from soil in New York state. See Ahmad and Baker (1987a,b) and Stasz et al. (1988).

Ahmad JS, Baker R. 1987a. Competitive saprophytic ability and cellulolytic activity of rhizosphere-competent mutants of *Trichoderma harzianum*. Phytopathology 77:358–362.

—, —. 1987b. Rhizosphere competence of *Trichoderma harzianum*. Phytopathology 77:182–189.

Stasz TE, Harman GE, Weeden NF. 1988. Protoplast preparation and fusion in 2 biocontrol strains of *Trichoderma harzianum*. Mycologia 80:141–150.

SUPLEMENTARY TABLE II. Fungal strains isolated from Lebanon from different ecological site.

Site	Fugus location	Genus (according to ITS)
**Site 1 ICARDA**	**Soil**	*Alternaria sp.* (x1)
		Aspergillus sp. (x11)
		Cladorrhinum sp. (x2)
		Talaromyces sp. (x1)
	**Root or stem**	*Alternaria sp.* (x2)
		Fusarium sp. (x5)
		Mucor sp. (x1)
		Stromatinia sp. (x2)
**Site 2 Tal_Amara**	**Soil**	*Aspergillus sp.* (x2)
		Penicillium sp. (x1)
	**Root or stem**	*Boeremia sp.* (x1)
		Mucor sp. (x1)
**Site 3 Doueir**	**Soil**	*Fusarium sp.* (x1)
		Trichoderma sp. (x1)

	Mucor sp. (x1)
**Root or stem**	*Fusarium sp.* (x3)
	Trichoderma sp. (x1)
	Macrophomina sp. (x1)
	Microdochium sp. (x1)
	Mucor sp. (x1)

2 Supplementary Figures

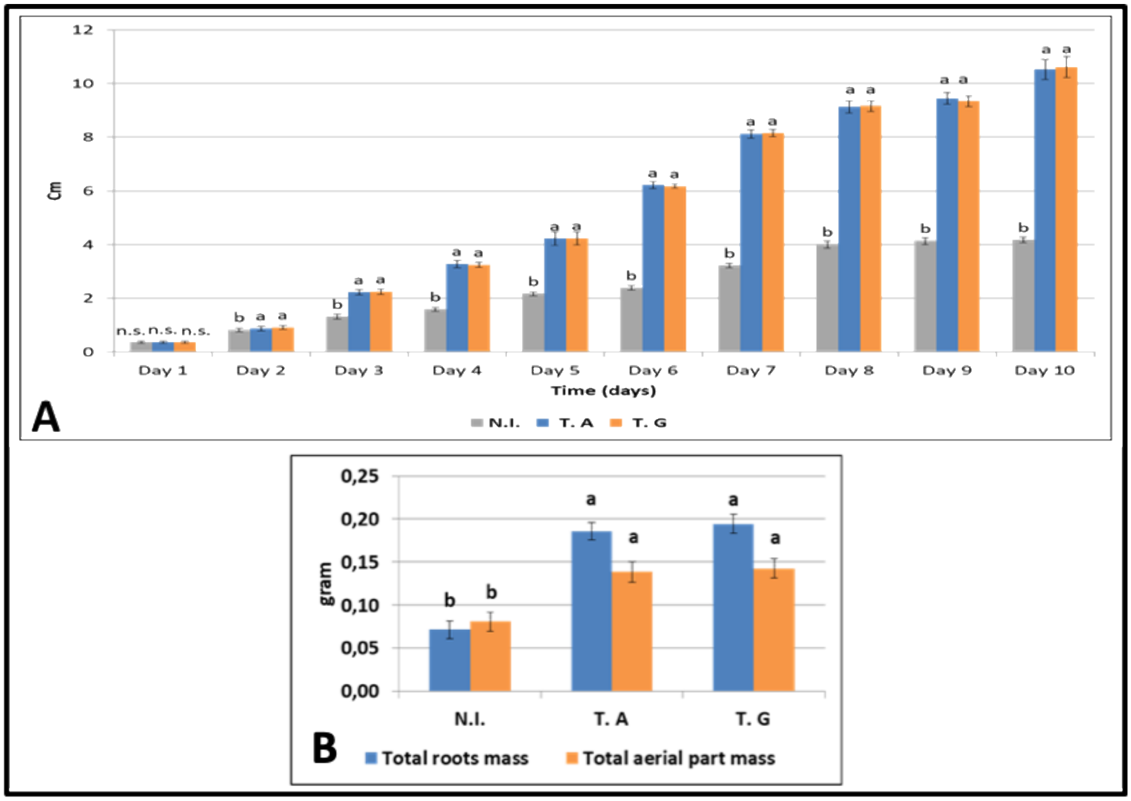

Supplementary Figure 1. (A) principal root length (cm) over a 10 days period. (B) total root, and aerial mass (g). N.I.: non-inoculated; T.A: wheat plants inoculated with *T. afroharzianum;* T.G: wheat plants inoculated with *T. guizhouense*. Values in the same column having different letters are significantly different, at P≤0.01 (ANOVA test).

.

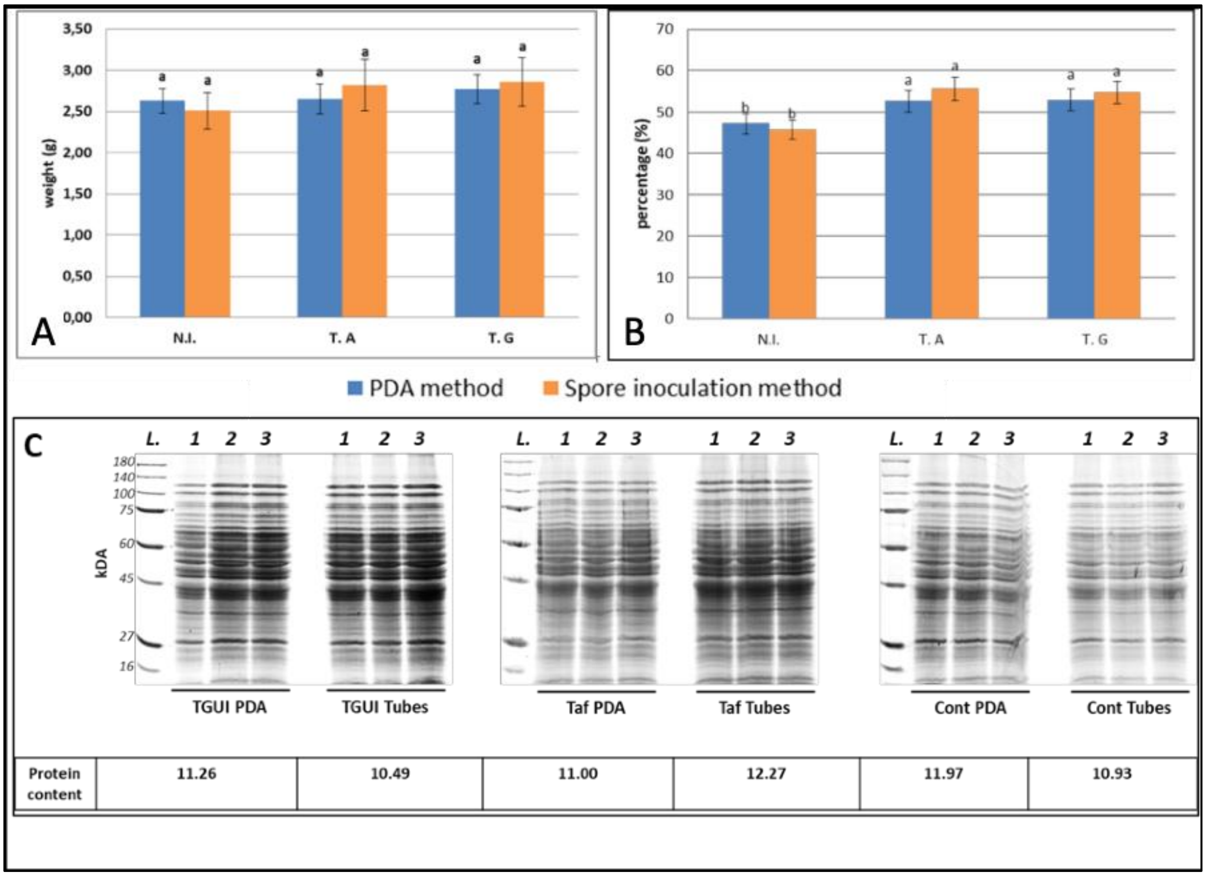

Supplementary Figure 2. (A) 100 grain weight (g) and (B) Harvest index (%). N.I.: non-inoculated; T.A: wheat plants inoculated with *T. afroharzianum*; T.G: wheat plants inoculated with *T. guizhouense*. (C) protein analysis (composition and content) of wheat plants non-inoculated (cont) or inoculated with *T. guizhouense* (TGUI) or *T. afroharzianum* (Taf) as influenced by PDA inoculation method (PDA) or the spore inoculation method (Tubes). The protein content of 100 grains in each condition is indicated below the gel pictures. Values in in each graph having different letters are significantly different, at P≤0.01 (ANOVA test).
